# Assessing the Efficacy of an Individualized Psychological Flexibility Skills Training Intervention App for Medical Student Burnout and Well-being: Protocol for a Randomized Controlled Trial

**DOI:** 10.2196/32992

**Published:** 2022-02-04

**Authors:** Elizabeth Ditton, Brendon Knott, Nicolette Hodyl, Graeme Horton, Frederick Rohan Walker, Michael Nilsson

**Affiliations:** 1 Centre for Rehab Innovations University of Newcastle Callaghan Australia; 2 Hunter Medical Research Institute New Lambton Heights Australia; 3 College of Health, Medicine and Wellbeing University of Newcastle Callaghan Australia; 4 Contextual Interventions Newcastle Australia; 5 Lee Kong Chian School of Medicine Nanyang Technological University Singapore Singapore

**Keywords:** burnout, psychological, burnout interventions, psychological flexibility, digital intervention, individualized intervention, acceptance and commitment therapy, medical students, well-being, mobile phone

## Abstract

**Background:**

Medical student burnout is a prevalent problem with adverse long-term outcomes. Incorporating psychological resource-building interventions into comprehensive burnout prevention approaches during medical training is an identified priority among educators. These interventions could reduce burnout risk by buffering students against nonmodifiable career stressors. However, there is a need for rigorous investigation into optimal intervention targets and methods. Psychological flexibility (PF) is an adaptive behavioral skill set that has demonstrated relationships with medical student burnout and well-being. More broadly, there is evidence that PF mediates burnout and well-being outcomes and may be a protective factor. Efficacy studies assessing the benefits of interventions targeting PF among medical students are needed. Research also supports the need to establish optimal methods for increasing intervention efficacy in the context of individual differences in burnout and PF by using individualized approaches.

**Objective:**

This study aims to assess whether an app-delivered PF intervention (Acceptance and Commitment Training) reduces burnout and improves well-being among medical students. We will examine whether changes in burnout and well-being are mediated by changes in PF. The potential benefits of an individualized version of the app versus those of a nonindividualized version will also be evaluated.

**Methods:**

In this 3-arm, parallel, randomized controlled study, a sample of medical students will be randomly allocated to 1 of 3 intervention arms (individualized, nonindividualized, and waiting list) by using a 1:1:1 allocation ratio. Participants in the individualized and nonindividualized intervention arms will have 5 weeks to access the app, which includes a PF concepts training session (stage 1) and access to short PF skill activities *on demand* (stage 2). Stage 2 will be either individualized to meet participants’ identified PF training needs at each log-in or nonindividualized.

**Results:**

Burnout, well-being, and PF will be assessed at baseline and after the intervention. Quantitative analyses will include descriptive and inferential statistics. We hypothesize that the Acceptance and Commitment Training intervention app will be effective in improving burnout and well-being and that changes in these outcomes will be mediated by changes in PF. We further hypothesize that participants in the individualized intervention group will demonstrate greater improvements in burnout and well-being outcomes than those in the nonindividualized group.

**Conclusions:**

The findings of this study could guide the development of burnout prevention and well-being initiatives for medical students. Identifying PF as a mediating process would provide support for the delivery of preventive intervention programs that train individuals to strengthen this psychological resource before burnout symptoms emerge. This would be an important step in addressing and potentially offsetting the significant costs of burnout among medical students and physicians. Demonstrating the superiority of an individualized version of the app over a nonindividualized version would have implications for enhancing intervention precision and efficacy by using scalable interventions.

**Trial Registration:**

Australian New Zealand Clinical Trials Registry ANZCTR 12621000911897; https://www.anzctr.org.au/ACTRN12621000911897.aspx

**International Registered Report Identifier (IRRID):**

PRR1-10.2196/32992

## Introduction

### A Burnout Epidemic

Burnout is a chronic and pervasive state of work-related stress that substantially disrupts an individual’s professional identity and psychological connections to their work [1]. The most widely accepted 3-factor model defines burnout as a psychological crisis that is experienced as a sense of fatigue, overload, and depleted emotional energy and resources (emotional exhaustion); a pattern of withdrawal behaviors; negative, detached attitudes toward others (cynicism); and the perception that one’s work performance lacks quality and value (inefficacy) [1,2]. Beyond the experience of burnout itself, affected individuals are at an increased risk of associated psychological difficulties [3-5], poor physical health outcomes [3,5-7], and cognitive impairments [8]. Through its impact on an individual’s health and capacity to work [9], burnout adversely affects organizational service delivery and productivity [5,10,11] and places a considerable burden on the economy [12].

Although burnout affects workers across a broad range of professional disciplines, it has become a problem of epidemic proportions within the field of medicine [11]. Prevalence estimates among physicians range between 37.9% [13] and 80.5% [14] compared with rates between 2% and 27.8% reported for the general working population [9,13]. Of particular concern is the increasing frequency of burnout experiences emerging during the early stages of medical training, with a global prevalence of approximately 44.2% among medical students [15]. Physicians and medical students who are affected by burnout experience higher risks of suicide [4,16-18], medical errors, and diminished quality of patient care [4,5,11,19,20].

### Study Aims

We aim to assess whether an app-based psychological skills training intervention is effective in reducing burnout and improving well-being among medical students, using research methodologies designed to maximize intervention precision and efficacy for individuals. In the following sections, we outline the background and rationale behind the selection of our intervention model, our methodology, and our research hypotheses.

### Calls for Early Intervention: Individual Psychological Resource Training

In response to the burnout crisis among physicians and medical students, there are increasing calls for the early implementation of interventions that facilitate the prevention of burnout and its broader associated outcomes [10,11,21-23]. Determining when and how to intervene most effectively remains an ongoing challenge. It has been proposed that burnout is an adverse psychological response to a pervasive imbalance between demands and individual coping resources, commencing during medical training, and persisting throughout a physician’s professional life [24]. Burnout interventions aim to minimize such imbalances, either by modifying external factors known to increase burnout risk at the organizational level or by training individuals to develop protective psychological and behavioral resources [25].

At an organizational level, learning institutions and workplaces may consider how to reduce modifiable external risk factors for burnout [23,26]. Risk factors that can and should be addressed in organizational settings include inadequate resources [27], excessive workloads, a lack of control or autonomy, a lack of reward, organizational unfairness [1], inflexible work schedules [23], bullying, and the mistreatment of students [28,29]. Interventions focused on minimizing organizational risk factors have demonstrated efficacy in reducing burnout among physicians [25]. However, physicians and students face many stressors that are inherent to their work [30], including exposure to distressing or demanding patient relationships, patient illness, death and dying, concerns about litigation, rapidly changing work and knowledge requirements, academic pressures, and performing highly responsible roles [4,20,24]. In a profession where such unmodifiable risk factors pervade, there is an important place for individual-focused cognitive and behavioral interventions. These interventions focus on enhancing modifiable individual psychological resources and adaptive coping skills [9] to buffer the potential adverse impacts of unmodifiable stressors and demands [9,30,31].

Importantly, although external factors contribute to burnout risk, medical students’ behavioral responses to stressors can also play a role [31]. For example, students who transition away from healthy behavior patterns during their medical training are at an increased risk of experiencing burnout [31] as are those who tend not to engage in values-driven behavior [32]. The risk is more than double for students who engage in maladaptive coping repertoires, such as avoidance-based coping (including cognitive, emotional, and behavioral avoidance) [32-35]. Longitudinal evidence suggests that medical students who demonstrate problematic coping patterns are unlikely to spontaneously adopt more effective behavioral repertoires over time [31], and this may have adverse consequences regarding long-term burnout and well-being outcomes [30]. Implementing interventions that promote the development of more adaptive coping repertoires could mitigate behavioral burnout risk factors during the early stages of an individual’s medical career [30].

Although organization-level interventions focus on removing contextual risk factors for burnout, individual interventions have the additional benefit of promoting behaviors that facilitate well-being [36,37], which is an identified goal within the medical field [29,38]. Well-being is more than just the absence of ill-health. Rather, it is a state of individual thriving that includes satisfaction with life, the experience of positive affect, engagement in values-aligned activities that promote purpose and vitality, and a sense of social connection that involves valuing and feeling valued by others [39]. It has been proposed that well-being interventions focusing on purposeful and valued living may be more important to individuals than those focusing solely on the removal of stressors [40]. Beyond the baseline goal of staving off burnout, individual interventions can empower medical students and physicians to build skills that facilitate the preservation and cultivation of sustainable well-being during their careers [5,14,41].

In light of these factors, the provision of individual psychological resource-building interventions as early as during undergraduate medical training is receiving growing support [18,20,22,29-31,33,38]. The past 10 years have seen a marked increase in the number of studies investigating such interventions [23]. A recent consensus statement issued by medical educators advocated for initiatives promoting “adaptive responses to stressful situations” as an important component of a broad well-being and burnout prevention plan for medical students in Australia and New Zealand [29]. Similarly, a recent review study identified the need for medical schools to foster training initiatives that ground students in the development of healthy habits, self-awareness skills, and effective mechanisms for coping with stressful experiences [18].

In nonmedical populations, meta-analyses [42,43] and systematic reviews [9,44] demonstrated that cognitive and behavioral interventions can be effective in reducing [9,42,43] and preventing [43,44] work-related stress and burnout. Recent systematic review findings provide support for the potential benefits of such interventions when delivered in medical training settings, however, there remain substantial knowledge gaps regarding the optimal methods for improving medical students’ well-being and reducing burnout risk [23]. The identification of the most effective intervention model and methods is constrained by the considerable heterogeneity between intervention approaches and the small number of randomized controlled trials (RCTs) within the existing research [23]. There is a need for further research adopting more “robust and rigorous” research methodologies as well as the use of “systematic and evidence-based” approaches when developing interventions [23].

Our randomized controlled study responds to the need for well-controlled research that can identify effective methods for mitigating the problem of burnout among physicians and medical students, using individual resource-building interventions during early medical training. In line with the identified research needs [23], we developed an app-delivered cognitive behavioral intervention that was grounded in the robust evidence-based psychological flexibility (PF) model. We outline the theoretical relevance of this model regarding these outcomes and this population and the importance of using rigorous mediational and individualized methodological approaches that can improve intervention precision.

### PF Model

#### The Theoretical Model

PF is a model of adaptive behavior that is rigorously grounded in behavioral science [45]. The model is composed of 6 modifiable behavioral flexibility skill sets or *processes* (and corresponding inflexibility processes) [46-48], including present-moment awareness (nonawareness of the present moment), experiential acceptance (experiential avoidance), cognitive defusion (cognitive fusion), self-as-context (self-as-content), contact with values (lack of contact with values), and committed action toward values (inaction) [47,49]. PF reflects the degree to which an individual is able to bring conscious awareness to a broad range of internal and external influences on their behavior and purposefully engage in actions directed toward personally held values and related goals [50]. When faced with challenges and stressors, people with high PF tend to choose adaptive behavioral responses that are driven by their values and facilitate well-being rather than behaviors that are rigidly driven by internal emotional and cognitive experiences [48,51]. PF and its individual processes are associated with a range of psychological health and well-being outcomes [51-53].

#### The Intervention: Acceptance and Commitment Training

PF processes are modifiable using cognitive behavioral training programs, such as Acceptance and Commitment Training (ACT; referred to as *ACT* in nonclinical settings and *Acceptance and Commitment Therapy* in clinical settings [45]). The benefits of ACT interventions are well established and have been demonstrated in over 600 RCTs [54]. Interventions focus on facilitating the development of behavioral repertoires that are more flexibly responsive to an individual’s present-moment experiences. This is achieved by training individuals to develop greater awareness of the influence of their internal experiences (eg, thoughts and emotions) on their actions, learning to alter their relationships with these internal experiences such that they are more accepting, and developing more flexible values-oriented behavioral repertoires [45].

#### PF as an Individual Protective Factor and Intervention Target

There is increasing evidence that PF skills can function as protective individual resources regarding burnout and well-being. Recent research has demonstrated associations between individual PF processes and work-related stress [39,41], burnout [55-58], and well-being [39,41,58,59]. PF processes account for unique variance in burnout symptomatology [41,60] and well-being [53]. These demonstrate predictive validity related to long-term workplace mental health [61] and show stronger and more consistent relationships with burnout and work-related well-being than do traditional organizational factors, such as job control and workload [41,56]. Interestingly, PF has also been shown to influence the degree to which employees are able to notice, engage with, and benefit from changes made to organizational burnout risk factors (eg, job control) [61], providing further support for the value of implementing individual interventions in conjunction with organizational interventions. PF skills have been shown to mediate relationships between stressors (eg, COVID-19–related stress) and well-being outcomes [62]. In nonmedical populations, interventions targeting individual components of the PF skill set demonstrate efficacy relating to reducing work-related stress and burnout [63-69], improving well-being [70], and improving coping in response to work-related stressors [63]. ACT interventions that provide training in the full PF skill set have been shown to reduce workplace stress and improve well-being [39].

#### Relevance of PF to Medical Students and Physicians

PF skills appear to function as important personal resources among medical students and physicians during the early stages of their careers [33]. Among medical students, lower PF is associated with reduced life satisfaction and greater personal distress when seeing others in harm, which may increase burnout risk [33]. Medical students who demonstrate behaviors associated with low PF (eg, avoidance and nonvalues-driven) are at greater risk of burnout. This leads to the suggestion that PF skills training could be of benefit to this population [32,33]. Similarly, burnout risk is higher among resident physicians with low PF [71]. Interventions targeting components of the PF model (eg, present-moment awareness and values) can effectively reduce stress and burnout risk among physicians and medical students [11,23,72]. To the best of our knowledge, no previous intervention studies have been found that have trained medical students or physicians in the full PF skill set.

### PF Training and Medical Student Burnout and Well-being

The literature has demonstrated associations between PF and burnout and well-being outcomes and evidence for the relevance of these processes to medical student and physician burnout and well-being. On this basis, we expect that training medical students to develop PF skills will have positive benefits concerning burnout and well-being. Given the absence of intervention studies with this population where all 6 PF processes are trained, we will assess the efficacy of our purpose-developed app-based ACT intervention for medical students. Stage 1 of the intervention will deliver a brief educational module that provides a conceptual explanation of each of the 6 PF processes as well as self-reflection and experiential skill practice activities.

Hypothesis 1 states the following: on the basis of the theoretical background presented, we hypothesize that medical students in the individualized and nonindividualized intervention groups will demonstrate significantly greater improvements in PF, burnout, and well-being outcomes (before and after intervention) compared with those in a waiting-list control group.

### PF as a Mechanism of Burnout and Well-being Change

One of the pitfalls of many cognitive behavioral intervention studies is that they deliver training packages that demonstrate efficacy regarding outcomes of interest, without identifying the mechanisms through which these interventions exert their effect [40]. Understanding the processes that underlie an intervention’s efficacy can facilitate greater intervention precision [40]. For example, knowing that PF is a mechanism of change for medical student burnout would open up opportunities for the delivery of other interventions that may improve these skills and facilitate the efficient delivery of the intervention *dose* needed to produce meaningful improvements in PF to reduce a particular individual’s burnout risk. Demonstrating the efficacy of an intervention through its impact on mediating processes is particularly important when seeking to prevent a distal adverse outcome. This provides opportunities to deliver interventions that strengthen these mediating skill sets among at-risk individuals (eg, medical students) before the emergence of adverse outcomes (eg, burnout).

Expectations of the efficacy of the current ACT intervention regarding burnout and well-being are based on the assumption that the training will help medical students use PF skills as an adaptive coping resource. This assumption is supported by previous mediation studies that demonstrated that ACT interventions influence burnout and well-being outcomes by improving an individual’s PF skills [39,58]. PF processes may also mediate longer-term burnout risk as demonstrated in intervention studies where improvements in PF processes reduced the subsequent risk of burnout development [55]. To assess the assumptions of our intervention and improve the rigor of our efficacy assessment, we will explore whether PF is a mechanism of change for any observed burnout and well-being intervention effects.

Hypothesis 2 states the following: on the basis of the theoretical background provided, we hypothesize that changes in burnout and well-being outcomes will be mediated by changes in PF.

### Individual Differences and Intervention Precision

There is a high degree of heterogeneity in both the way burnout symptoms develop among individuals over time [36] and individual recovery patterns during and after burnout interventions [9,36,69]. Similarly, although some individuals demonstrate reasonably consistent patterns of high or low PF across all processes, a range of distinct and more complex profiles of flexibility and inflexibility have recently been observed [47,48,53,60]. Each of these unique PF profiles demonstrates different relationships with a range of ill-being and well-being outcomes [47,48,53]. These findings demonstrate that individuals might require different types of interventions that target their individual risk factors to experience improvements in burnout risk and well-being outcomes [36,37,47,69,73]. This poses problems for static interventions that provide the same training for all individuals, as it means that some may not receive the type or amount of training they need, and others may receive more training than necessary [40,69]. Furthermore, interventions that are highly effective for some individuals, but not others, might not be further developed and disseminated because they fail to demonstrate efficacy at the group level [69].

As such, there are increasing calls by leading researchers for the use of individualized intervention methodologies, whereby an intervention is tailored to target the identified needs of each individual [40]. Individualized interventions can be delivered with a higher degree of precision than static interventions, providing the potential for superior efficacy and efficient resource allocation [74]. To address individual-level differences in this study, we will adopt a *treatment utility* methodology that compares an individualized version of the PF skills training app with a nonindividualized version [40]. Stage 1 of the intervention will deliver a static PF training module, and stage 2 will provide access to a library of short PF skills training activities that participants can access *on demand*. Participants receiving the individualized intervention will practice activities from the PF skill set that aligns with their identified needs during each training session, whereas those receiving the nonindividualized intervention will receive nontargeted training from any of the PF skill sets. This approach addresses individual differences in PF at the intervention level, allowing us to compare burnout and well-being outcomes between individualized and nonindividualized groups. A recent study using a similar methodological design demonstrated that an individualized version of a PF intervention was more effective than a nonindividualized version in reducing general psychological distress and improving mental health outcomes [75]. We expect that an individualized intervention will address the problem of individual heterogeneity by delivering more relevant and precisely targeted intervention skills than the nonindividualized version.

Hypothesis 3 states the following: we hypothesize that medical students in the individualized intervention group will demonstrate significantly greater improvements in PF, burnout, and well-being outcomes (before and after intervention) than those in the nonindividualized group.

### App-Based Intervention Delivery

Review studies of individual resource-building interventions for medical students highlight the need to explore the efficacy of delivering interventions using nontraditional methods, (eg, smartphone apps) [23]. To the best of our knowledge, there are no published studies assessing the efficacy of smartphone app-based cognitive behavioral interventions for burnout or well-being among medical students or physicians. We adopted this mode of delivery for 5 main reasons. First, app-based platforms facilitate the tailoring of interventions to the needs of each participant, providing opportunities to deliver targeted skills training activities appropriate to the individual’s needs in a particular moment [75]. Second, research indicates that medical students tend not to seek support for burnout owing to the perceived stigma related to mental health and beliefs that others will see them as weak or unable to cope with working in the field [24,34]. App-based interventions have the potential to provide greater acceptability to students with these concerns by allowing them to maintain discretion, anonymity, and privacy while engaging in an intervention [34]. Third, the time requirements of participating in well-being programs can also be a barrier to engagement among medical students [76]. App-based formats facilitate the delivery of short training activities and flexible completion options, which may buffer the impact of time-related barriers [34]. Fourth, there is evidence supporting the acceptability and efficacy of web-based PF interventions among university students as well as the capacity for such interventions to promote intervention engagement [75]. Finally, app-based interventions can be favorable to stakeholders because of their scalability, making them accessible to large numbers of individuals [18]. This is particularly important in circumstances where access to health care personnel is limited or where face-to-face interventions are not practical or possible (eg, during a global pandemic).

## Methods

### Study Setting

This study will be conducted with the first-, second-, fourth-, and fifth-year undergraduate medical students enrolled in the Joint Medical Program (JMP) at the University of Newcastle and University of New England, Australia, which included 778 enrolled students in June 2021.

### Study Design

This study will adopt a 3-arm, parallel, partially blinded (participants), randomized controlled design.

**Figure 1:**
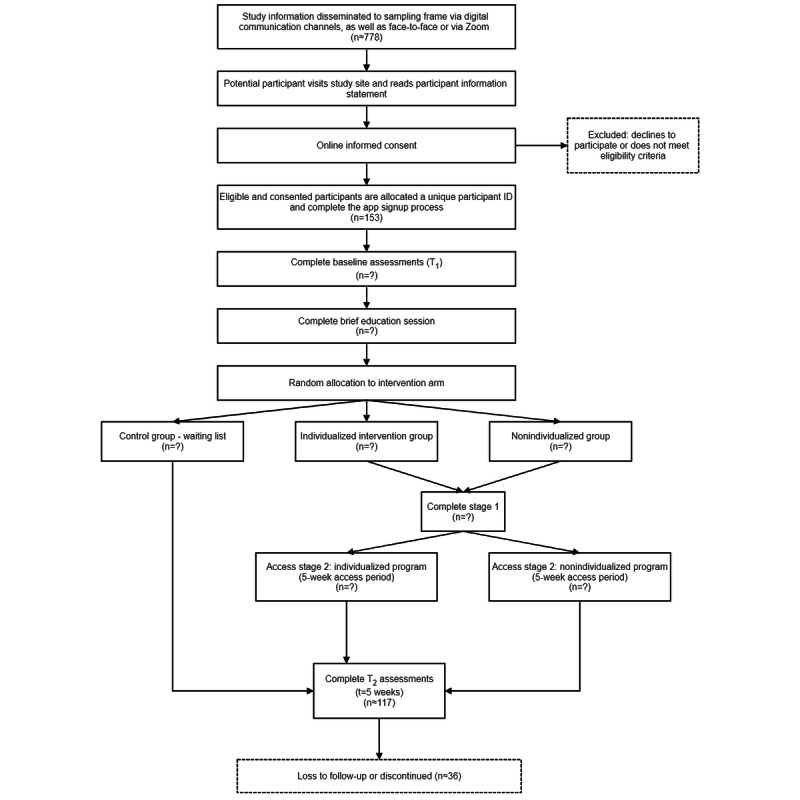
Participant timeline.

All participants will complete a brief educational session that introduces the potential benefits of PF skills relating to burnout and well-being. Following this, they will be randomized into 1 of the 3 intervention arms using a 1:1:1 allocation ratio. Randomization will be conducted within the app. The three intervention arms are as follows: (1) individualized intervention, (2) nonindividualized intervention, and (3) waiting list. Figure 1 outlines the participants’ timeline for this study.

### Sample Size

We conducted a power analysis to determine an appropriate sample size for a repeated measures multivariate analysis of variance (3 groups; measuring 2 time points [baseline and after the intervention]; high correlation between pre- and postintervention measures, 0.6). The calculation allowed for a small but still clinically meaningful effect size (0.25), given that this is the most conservative approach. For power of 80% and Cronbach α of .05, the total required sample size is 117 (39 per intervention arm). We allow for an attrition rate of up to 30% and set our target recruitment sample size at 153 participants (51 per intervention arm).

### Eligibility

Participants will be eligible and included if they are enrolled in the first, second, fourth, or fifth year of the JMP at the University of Newcastle or enrolled in the first, second, fourth, or fifth year of the JMP at the University of New England and have regular access to a reliable internet connection and have regular access to an electronic device that is compatible with app use (smartphone or tablet). Third-year students will not be recruited for this trial, as this cohort was previously invited to participate in a pilot trial of the app intervention.

### Recruitment and Enrollment Procedures

Eligible students will be invited to participate in the study via the universities’ digital media and communication channels, including internal departmental mailing lists and e-newsletters, blackboard sites, and brief information sessions presented verbally by a researcher who is not connected with the JMP. Participation will be voluntary.

Students who are interested in participating will access the enrollment and consent page using a URL or QR code. The eligibility, consent, and enrollment processes will be conducted using REDCap (Research Electronic Data Capture; Vanderbilt University). Eligible participants should read the participant information statement on the web. Those who elect to participate will provide their consent via e-consent. Once consented and enrolled, participants will complete a brief demographic questionnaire, following which they will be assigned a unique participant ID and receive instructions for downloading the app. The app will be available for download through the App Store (Apple) and Play Store (Android).

### Data Collection Tools and Procedures

Data will be collected at two time points: T_1_ (baseline) and T_2_ (following the completion of the app-based intervention, commencing 5 weeks after baseline).

Study data will be collected via the app, which will record participant responses to questionnaires as well as app use data. Table 1 shows the administration time points for each outcome measure.

**Table 1:** Outcome measures and administration time points.

Outcome	Assessment	Baseline (T_1_)	After the intervention (T_2_)	Every log-in (stage 2)
Demographics	Demographics	✓		
Burnout	Maslach Burnout Inventory–General Survey for Students	✓	✓	
Well-being	Mental Health Continuum–Short Form	✓	✓	
Psychological flexibility and psychological inflexibility	Multidimensional Psychological Flexibility Inventory–Short Form	✓	✓	
Depression, anxiety, and stress	Depression Anxiety and Stress Scale-21	✓	✓	
Current psychological flexibility difficulty	Check-in assessment			✓
Activity acceptability	Like or dislike			✓

### Demographic Information

The following demographic information will be collected from the participants: age, gender, university enrollment status, indigenous status, work history, previous history of burnout, current engagement in psychological therapy or other treatment, and self-rating (5-point Likert scale) of current physical health, diet, and self-care behavior.

### Primary Outcome: Burnout

We will assess burnout using the 3-factor Maslach Burnout Inventory–General Survey for Students [77]. This 16-item self-report questionnaire assesses the degree to which participants are experiencing each of the following burnout components: *Exhaustion,* stress; *Cynicism,* attitude; and *Academic Inefficacy,* achievement. The Maslach Burnout Inventory scale is valid [2], and the reliability of the Maslach Burnout Inventory–General Survey for Students has been demonstrated among a sample of medical students from the United States [76].

### Secondary Outcome: Well-being

Reducing burnout risk via the enhancement of PF processes is the primary goal of the skill development program, however, it is also known that PF tends to improve well-being outcomes. This is an important goal because psychological health is not only reflected by the absence of adverse outcomes but also by the presence of well-being [78]. A secondary goal of the intervention program is to facilitate improved well-being among the participants. We will assess well-being using the Mental Health Continuum–Short Form [78]. This 14-item self-report questionnaire assesses the frequency of well-being experiences during the previous month. The scale has demonstrated validity [78], reliability [75,79], and sensitivity to changes in web-based intervention studies [80].

### Process Outcome: PF

We will assess PF and psychological inflexibility (PI) using the Multidimensional PF Inventory–Short Form [47]. This 24-item self-report questionnaire assesses the frequency of each PF and PI experience during the previous 2 weeks. In addition to global PF and PI scores, individual subscale scores can be calculated for each of the 6 PF and 6 PI processes. The scale has demonstrated validity [53] and reliability [81] and is responsive to changes over time [47].

### Depression, Anxiety, and Stress

Depressive symptoms, anxiety, stress, and general negative affectivity will be screened as secondary outcomes for two key reasons: (1) for comparison purposes with previous similar studies and (2) to explore whether these factors are related to engagement in the intervention as has been observed in previous studies [82]. We will assess these symptoms using the Depression Anxiety and Stress Scale-21 [83]. This valid and reliable 21-item self-report questionnaire assesses the degree of symptoms of depression, anxiety, and stress during the previous week and a general negative affectivity factor [84].

### Engagement Data

We will assess the following behavioral engagement metrics using app use data.

#### Recruitment

The number of participants who enroll in the study during the recruitment period will be assessed.

#### Adherence

The proportion of recruited participants who meet adherence requirements, defined as the completion of stage 1 and engagement in at least four skill activities during stage 2 (individualized vs nonindividualized analyses only), will be assessed.

#### Attrition

We will assess attrition at all stages of the program: enrolled but did not sign-up to use the app, did not complete T_1_ assessments, did not complete brief educational module, did not complete stage 1, did not complete adherence requirements for stage 2 (individualized vs nonindividualized analyses only), and did not complete T_2_ assessments.

#### App Engagement Frequency

The frequency of participant engagement with components of the app during stage 2 will be assessed using the following: app log-ins, check-in assessments completed, skill activities completed, and skill activities completed per log-in.

#### Individual Skill Activity Feedback

Following the completion of each stage 2 skill activity, participants will be asked to indicate whether they *liked* the activity by selecting either a thumbs-up (like) or thumbs-down (dislike) icon.

### Intervention

#### PF Model: ACT Intervention

This study will deliver an ACT PF skills training intervention in written and audio formats via a web-based app. ACT facilitates the development of individual PF skills for nonclinical populations, promoting and facilitating actions that support individual well-being [52,67]. Rather than defining uncomfortable internal experiences (eg, thoughts, emotions, and physical sensations) as *symptoms* that need to be changed or eliminated, ACT focuses on altering the way individuals relate to these internal experiences [46,85] and empowering them to respond in ways that are more adaptive in their own lives [86]. This is accomplished through activities that normalize challenging internal experiences as well as training individuals to strengthen present-moment awareness, recognize the influence of their internal experiences over their actions, learn to alter unworkable responses to these internal experiences, and develop more flexible behavioral repertoires that are consistent with their personally chosen values [45,46,85,86].

ACT is conceptually well defined and links its interventions to the mechanisms through which it purportedly elicits change (ie, the 6 PF processes and their corresponding inflexibility processes) [87]. The components of an ACT intervention include the following: present-moment awareness (mindfulness), cognitive defusion, self-as-context, acceptance, values, and committed action.

Two clinical psychologists (first and second authors: ED and BK) with extensive training and experience with ACT interventions and the PF model designed the app intervention. The piloting of the intervention among a small cohort of medical students at the University of Newcastle indicated high acceptability and usability.

#### Intervention Stages

All participants will complete a brief introductory session that provides education about burnout, psychological well-being, and PF skills (approximately 10 minutes). Participants will then be randomly allocated to the waiting list, individualized intervention, or nonindividualized intervention arm. Participants who are allocated to the individualized and nonindividualized groups will have access to the 2-stage app for 5 weeks.

Stage 1 (Learn the Concepts) presents a conceptual overview of each PF process and provides a framework through which participants can understand the rationale and potential personal relevance of the skills they will be practicing in stage 2. During stage 1, participants are given opportunities to practice each PF skill set. Depending on whether participants choose to complete these optional skill activities, the completion time for stage 1 is approximately 30 to 60 minutes and can be completed over more than one sitting. The completion of stage 1 will unlock access to stage 2.

During stage 2 (Learn the Behaviors), participants will have access to *on demand* PF skills training. They will be asked to complete a check-in assessment each time they access the app, to assess which PF process they are currently experiencing the most difficulty with. For the individualized intervention arm, a participant’s check-in assessment response at each log-in will be used to individualize their intervention. That is, participants will receive access to training in whichever skill they report having the most difficulty with in that moment. Participants will be directed to a dashboard that gives them access to all the available skill activities for their identified PF process (eg, if a participant identifies that they are having difficulty attending to the present moment, they will be presented with the present-moment awareness dashboard). Participants may then select an activity or have the app select a random activity for them from within the targeted PF skill set. This method is similar to one adopted in a recent study assessing a web-based ACT intervention for anxiety and depression [75]. For the nonindividualized intervention arm, check-in assessment responses *will not* be used to select a targeted PF skill set for the participant’s training during that session. Rather, participants will be directed to any of the PF process dashboards at random.

Upon the completion of a stage 2 skill activity, participants will be asked if they would like to complete another activity. If they select *yes,* those in the individualized group will return to the dashboard of the PF process identified at check-in and may repeat the aforementioned activity selection process. Participants in the nonindividualized group will be presented with another random activity. Participants may complete as many activities as they choose, but will be asked to complete at least four stage 2 skill activities during their 5-week period of access to the app.

Each of the 6 PF dashboards includes 20 brief (2-7 minutes) experiential skill-building activities, resulting in a total of 120 activities available in stage 2. The inclusion of this number of activities was guided by previous studies, indicating that 28 activities were too few [88] and 136 activities were sufficient [75]. An example activity for each skill set is provided in Multimedia Appendix 1, along with the activity aims as they relate to the PF processes.

### App Engagement

Previous piloting of this intervention by our research team indicated that self-reported acceptability by medical students did not necessarily correspond with regular behavioral engagement with the app. Pilot findings showed high engagement in stage 1 but lower than expected engagement in stage 2. Facilitating ongoing user engagement is a known challenge for developers of app-based mental health interventions [89]. The current version of the intervention incorporates the pilot study participants’ feedback and suggestions to enhance engagement for the RCT. This includes ensuring that participants are clear about what to expect throughout the intervention, providing clear indicators of progress and achievement, providing opportunities for personally relevant interactive learning and self-reflection, delivering in-app reminder notifications (push notifications), and delivering information in multiple forms where possible (eg, written and audio) to accommodate individual learning preferences and situational conditions. The app will also encourage engagement through the provision of positive reinforcement when a participant completes certain activities or completes a particular number of activities (eg, an achievement badge acknowledging their commitment and the benefits of practice).

### Data Analysis

Descriptive and inferential statistics will be used to describe demographic, outcome, and app engagement data. To assess hypotheses 1 to 3, modeling to examine treatment efficacy with follow-up outcomes will be performed, using mixed models (with appropriate distribution and link function), including a random intercept for participants to account for repeated measures. Descriptive analyses will be performed for use data obtained from the app platform to assess engagement, including engagement frequency and retention and adherence rates.

### Institutional Review Board Approval

This research methodology was peer reviewed and approved by the School of Medicine and Public Health at the University of Newcastle, in accordance with the Australian Code for the Responsible Conduct of Research. The study will be conducted according to the National Health and Medical Research Council National Statement on Ethical Conduct in Human Research (2007). Ethics approval was granted by the University of Newcastle Human Research Ethics Committee (approval ID: H-2020-0311) on July 13, 2021, and ratified by the University of New England Human Research Ethics Committee.

## Results

Recruitment for this study occurred between August 2021 and September 2021. Data collection for this sample was completed during December 2021. The study results will be available within 12 months of the final data collection date (ie, results are expected to be available by late 2022). The findings will be disseminated to stakeholders by using various methods, including the lead author’s doctoral dissertation, peer-reviewed journals, academic conferences, and other verbal and digital communication channels.

## Discussion

### Overview

The main aim of this study is to assess whether an app-based ACT intervention is effective in reducing burnout and improving well-being among medical students. On the basis of the literature outlined in the *Introduction*, we expect that students in the intervention groups will demonstrate improved burnout and well-being outcomes compared with students in the waiting-list control group. We will also examine whether changes in burnout and well-being are mediated by PF, which is the hypothesized mechanism of change targeted by the intervention. We will further evaluate whether an individualized version of the intervention is more effective than a nonindividualized version. The individualized version focuses on delivering PF skill activities that specifically target individual participants’ needs each time they access the app; therefore, we hypothesize that this version will be more effective in improving all outcomes than the nonindividualized version.

This study responds to an identified need for the development of early intervention initiatives that could prevent burnout and improve well-being among medical students by targeting modifiable individual risk factors [20,22,29-31,33,38]. Although there has been an increase in the number of studies assessing such interventions in recent years [23], there is a need for more rigorous approaches that can clarify optimal intervention targets and methods [23,90]. We contribute to existing research by delivering a PF intervention based on sound principles of behavioral science, adopting a robust randomized controlled design, assessing mediators of changes in outcomes, and addressing heterogeneity among individuals by assessing the potential additive benefits of an individualized intervention approach. Our intervention builds on existing research demonstrating the involvement of PF processes as predictors, moderators, and mediators of burnout and well-being outcomes [11,55-58,63-67,69,70,72]. There is evidence that PF is an individual resource of relevance to burnout and well-being among medical students and physicians [23,32,33,71,72]; however, we are unaware of any published RCTs assessing the benefits of training the full PF skill set within this population to date. To the best of our knowledge, this is also the first individualized app-based skills training intervention targeting burnout prevention and well-being among medical students.

### Contributions and Theoretical Implications

The demonstration of full or partial support for our hypotheses will provide important information regarding the strategies for improving burnout and well-being during undergraduate medical training. If the ACT intervention demonstrates efficacy regarding burnout and well-being outcomes, this would strengthen the evidence base for PF as a modifiable psychological resource of importance among medical students. It would also be the first study to demonstrate the benefits of providing PF skills training to this group. This finding would also contribute to the growing body of literature supporting the general role of PF skills in maintaining a healthy career [55-58,63-67,69,70,72]. Furthermore, demonstrating that the intervention exerted its effect on outcomes via its capacity to improve individual PF processes would have important implications for burnout prevention research. If PF is identified as a mediator of burnout and well-being outcomes among medical students, future interventions could specifically target PF skills as a preventive measure. This approach could offset the risk of burnout *before* symptoms emerge and proactively improve well-being. The identification of PF as a mediating process would provide a metric for assessing an individual’s potential degree of burnout risk and their responsiveness to a preventive intervention.

The study findings could also have implications for intervention precision. This is important for stakeholders seeking to deliver initiatives as effectively and efficiently as possible, and medical students seeking time-efficient and personally relevant training [76]. Identifying mediators of burnout and well-being outcomes could facilitate the precise delivery of only the training components necessary to generate changes in the mediating processes, allowing for the elimination of extraneous components [37,40]. Findings regarding the potential additive benefits of individualized intervention have further implications for intervention precision. Static interventions experience the problem of delivering the same training components to all participants, without sensitivity to the differences in individual training needs [37,40]. Previous research has demonstrated the benefits of individualized PF interventions over nonindividualized versions regarding other outcomes (eg, depression and anxiety [75]). Replicating these findings for burnout and well-being outcomes would further support the importance of accounting for individual differences when developing interventions and demonstrate an effective method for doing so.

Finally, the delivery of this intervention via an app is a unique approach, and to the best of our knowledge, there are no published RCTs assessing an intervention of this nature with medical students. The demonstration of the efficacy of an app-based intervention for medical student burnout and well-being provides a potential solution to the problem of delivering well-being resources to this group. App-based interventions address a range of challenges by offering accessibility, privacy, time efficiency, precision, and scalability. These findings are important to both organizational stakeholders and medical students seeking strategies for building sustainable work-related well-being.

### Limitations

Although the study represents a rigorous and robust contribution to the existing literature, there are potential limitations. This study will assess the outcomes at baseline and following the completion of the intervention. This may not allow sufficient time for burnout or the well-being–related benefits of the intervention to manifest. As such, we intend to conduct subsequent follow-up studies with consenting participants to assess the long-term outcomes. As previously noted, early piloting of the intervention highlighted engagement issues regarding stage 2 of the intervention. Although we believe we have rectified these issues based on feedback from the pilot study, it is possible that participant engagement in stage 2 might be limited. This would hinder our efforts to assess the differences between individualized and nonindividualized versions. Additionally, the problem that we are seeking to address could further hinder engagement in this study, with stressed students potentially being less likely to maintain their participation over time. Early piloting suggests that short intervention components are a positive factor in facilitating intervention engagement among medical students but that time constraints might adversely impact the completion rates of postintervention outcome measures.

We anticipate that the intervention will produce positive outcomes, but there is a risk of the misuse of such findings. As previously noted, interventions empowering students and physicians to build psychological well-being should be incorporated into broader prevention initiatives that take steps to mitigate modifiable external burnout risk factors. However, organizations might overemphasize the role of individual interventions, placing the burden of burnout prevention solely on medical students and physicians. We advocate against the use of this type of training as a stand-alone intervention but rather as a component of a broader strategy that includes organizational-level changes where needed [29].

### Conclusions

This study will contribute to the existing literature aiming to elucidate effective methods for improving medical student well-being and preventing burnout and identifying whether PF is a worthy target of future interventions. We offer a unique method of intervention delivery that can deliver individualized skills training with precision, in a way that is acceptable and scalable among medical student populations. This research has the potential to provide a strong foundation upon which to establish preventive resources and interventions for medical students and professionals, which could offset the broader consequences of burnout for individuals, organizations, patients, and the global economy.

## Appendix

Multimedia Appendix 1 Aims and example activities for each psychological flexibility skill set.

## Abbreviations

ACT: Acceptance and Commitment Training
JMP: Joint Medical Program
PF: psychological flexibility
PI: psychological inflexibility
RCT: randomized controlled trial
REDCap: Research Electronic Data Capture

## References

[1] Leiter MP, Maslach C (2016). Latent burnout profiles: a new approach to understanding the burnout experience. Burnout Res.

[2] Maslach C, Jackson SE, Leiter MP (1996). Maslach Burnout Inventory Manual.

[3] Kishita N, Shimada H (2011). Effects of acceptance-based coping on task performance and subjective stress. J Behav Ther Exp Psychiatry.

[4] Kumar S (2016). Burnout and doctors: prevalence, prevention and intervention. Healthcare (Basel).

[5] Maslach C, Leiter MP (2016). Understanding the burnout experience: recent research and its implications for psychiatry. World Psychiatry.

[6] Andel R, Crowe M, Hahn EA, Mortimer JA, Pedersen NL, Fratiglioni L, Johansson B, Gatz M (2012). Work-related stress may increase the risk of vascular dementia. J Am Geriatr Soc.

[7] Schnall PL, Landsbergis PA, Baker D (1994). Job strain and cardiovascular disease. Annu Rev Public Health.

[8] Deligkaras P, Panagopoulou E, Montgomery AJ, Masoura E (2014). Job burnout and cognitive functioning: a systematic review. Work Stress.

[9] Ahola K, Toppinen-Tanner S, Seppänen J (2017). Interventions to alleviate burnout symptoms and to support return to work among employees with burnout: systematic review and meta-analysis. Burnout Res.

[10] Salvagioni DA, Melanda FN, Mesas AE, González AD, Gabani FL, Andrade SM (2017). Physical, psychological and occupational consequences of job burnout: a systematic review of prospective studies. PLoS One.

[11] West CP, Dyrbye LN, Erwin PJ, Shanafelt TD (2016). Interventions to prevent and reduce physician burnout: a systematic review and meta-analysis. Lancet.

[12] Hassard J, Teoh KR, Visockaite G, Dewe P, Cox T (2018). The cost of work-related stress to society: a systematic review. J Occup Health Psychol.

[13] Shanafelt TD, Kaups KL, Nelson H, Satele DV, Sloan JA, Oreskovich MR, Dyrbye LN (2014). An interactive individualized intervention to promote behavioral change to increase personal well-being in US surgeons. Ann Surg.

[14] Rotenstein LS, Torre M, Ramos MA, Rosales RC, Guille C, Sen S, Mata DA (2018). Prevalence of burnout among physicians: a systematic review. J Am Med Assoc.

[15] Frajerman A, Morvan Y, Krebs M, Gorwood P, Chaumette B (2019). Burnout in medical students before residency: a systematic review and meta-analysis. Eur Psychiatry.

[16] Kuhn CM, Flanagan EM (2017). Self-care as a professional imperative: physician burnout, depression, and suicide. Can J Anaesth.

[17] Dyrbye LN, Thomas MR, Massie FS, Power DV, Eacker A, Harper W, Durning S, Moutier C, Szydlo DW, Novotny PJ, Sloan JA, Shanafelt TD (2008). Burnout and suicidal ideation among U.S. medical students. Ann Intern Med.

[18] Harvey SB, Epstein RM, Glozier N, Petrie K, Strudwick J, Gayed A, Dean K, Henderson M (2021). Mental illness and suicide among physicians. Lancet.

[19] Shanafelt TD, Boone S, Tan L, Dyrbye LN, Sotile W, Satele D, West CP, Sloan J, Oreskovich MR (2012). Burnout and satisfaction with work-life balance among US physicians relative to the general US population. Arch Intern Med.

[20] Bugaj T, Cranz A, Junne F, Erschens R, Herzog W, Nikendei C (2016). Psychosocial burden in medical students and specific prevention strategies. Ment Health Prevent.

[21] Demerouti E (2015). Strategies used by individuals to prevent burnout. Eur J Clin Invest.

[22] Costa EF, Santos SA, Santos AT, Melo EV, Andrade TM (2012). Burnout Syndrome and associated factors among medical students: a cross-sectional study. Clinics.

[23] Seo C, Corrado M, Fournier K, Bailey T, Haykal K (2021). Addressing the physician burnout epidemic with resilience curricula in medical education: a systematic review. BMC Med Educ.

[24] Fares J, Al Tabosh H, Saadeddin Z, El Mouhayyar C, Aridi H (2016). Stress, burnout and coping strategies in preclinical medical students. N Am J Med Sci.

[25] De Simone S, Vargas M, Servillo G (2021). Organizational strategies to reduce physician burnout: a systematic review and meta-analysis. Aging Clin Exp Res.

[26] D'Eon MF, Thompson G, Stacey A, Campoli J, Riou K, Andersen M, Koehncke N (2021). The alarming situation of medical student mental health. Can Med Educ J.

[27] Bailey JG, Wong M, Bailey K, Banfield JC, Barry G, Munro A, Kirkland S, Leiter M (2021). Pandemic-related factors predicting physician burnout beyond established organizational factors: cross-sectional results from the COPING survey. Psychol Health Med.

[28] Dyrbye LN, Satele D, West CP (2021). Association of characteristics of the learning environment and us medical student burnout, empathy, and career regret. JAMA Netw Open.

[29] Kemp S, Hu W, Bishop J, Forrest K, Hudson JN, Wilson I, Teodorczuk A, Rogers GD, Roberts C, Wearn A (2019). Medical student wellbeing - a consensus statement from Australia and New Zealand. BMC Med Educ.

[30] Ishak W, Nikravesh R, Lederer S, Perry R, Ogunyemi D, Bernstein C (2013). Burnout in medical students: a systematic review. Clin Teach.

[31] Voltmer E, Rosta J, Aasland OG, Spahn C (2010). Study-related health and behavior patterns of medical students: a longitudinal study. Medical Teacher.

[32] Kroska EB, Calarge C, O’Hara MW, Deumic E, Dindo L (2017). Burnout and depression in medical students: relations with avoidance and disengagement. J Context Behav Sci.

[33] Palladino CL, Ange B, Richardson DS, Casillas R, Decker M, Gillies RA, House A, Rollock M, Salazar WH, Waller JL, Zeidan R, Stepleman L (2013). Measuring psychological flexibility in medical students and residents: a psychometric analysis. Med Educ Online.

[34] Thompson G, McBride RB, Hosford CC, Halaas G (2016). Resilience among medical students: the role of coping style and social support. Teach Learn Med.

[35] Arif NM, Roslan NS, Ismail SB, Nayak RD, Jamian MR, Roshidi AS, Edward TC, Kamal MA, Amin MM, Shaari S, Basri MF (2021). Prevalence and associated factors of psychological distress and burnout among medical students: findings from two campuses. Int J Environ Res Public Health.

[36] Mäkikangas A, Kinnunen U (2016). The person-oriented approach to burnout: a systematic review. Burnout Res.

[37] Hofmann SG, Hayes SC (2019). The future of intervention science: process-based therapy. Clin Psychol Sci.

[38] Dunn LB, Iglewicz A, Moutier C (2008). A conceptual model of medical student well-being: promoting resilience and preventing burnout. Acad Psychiatry.

[39] Wersebe H, Lieb R, Meyer AH, Hofer P, Gloster AT (2018). The link between stress, well-being, and psychological flexibility during an Acceptance and Commitment Therapy self-help intervention. Int J Clin Health Psychol.

[40] Hayes SC, Hofmann SG, Stanton CE, Carpenter JK, Sanford BT, Curtiss JE, Ciarrochi J (2019). The role of the individual in the coming era of process-based therapy. Behav Res Ther.

[41] Puolakanaho A, Tolvanen A, Kinnunen SM, Lappalainen R (2018). Burnout-related ill-being at work: associations between mindfulness and acceptance skills, worksite factors, and experienced well-being in life. J Context Behav Sci.

[42] Maricuţoiu LP, Sava FA, Butta O (2014). The effectiveness of controlled interventions on employees’ burnout: a meta-analysis. J Occup Organ Psychol.

[43] Ruotsalainen JH, Verbeek JH, Mariné A, Serra C (2015). Preventing occupational stress in healthcare workers. Cochrane Database Syst Rev.

[44] Awa WL, Plaumann M, Walter U (2010). Burnout prevention: a review of intervention programs. Patient Educ Couns.

[45] Moran DJ (2010). ACT for leadership: using acceptance and commitment training to develop crisis-resilient change managers. Int J Behav Consult Ther.

[46] Hayes SC, Strosahl KD, Wilson KG, Sandoz EK, Craske MG (2016). Acceptance and Commitment Therapy: The Process and Practice of Mindful Change.

[47] Rolffs JL, Rogge RD, Wilson KG (2018). Disentangling Components of Flexibility via the Hexaflex Model: Development and Validation of the Multidimensional Psychological Flexibility Inventory (MPFI). Assessment.

[48] Stabbe OK, Rolffs JL, Rogge RD (2019). Flexibly and/or inflexibly embracing life: identifying fundamental approaches to life with latent profile analyses on the dimensions of the Hexaflex model. J Context Behav Sci.

[49] Hayes SC, Pistorello J, Levin ME (2012). Acceptance and Commitment Therapy as a unified model of behavior change. Counsel Psychol.

[50] Hayes SC, Strosahl KD, Wilson KG (1999). Acceptance and Commitment Therapy: An Experiential Approach to Behavior Change.

[51] Onwezen MC, van Veldhoven MJ, Biron M (2012). The role of psychological flexibility in the demands–exhaustion–performance relationship. Eur J Work Organ Psychol.

[52] Biglan A, Hayes SC, Pistorello J (2008). Acceptance and commitment: implications for prevention science. Prev Sci.

[53] Rogge RD, Daks JS, Dubler BA, Saint KJ (2019). It's all about the process: examining the convergent validity, conceptual coverage, unique predictive validity, and clinical utility of ACT process measures. J Context Behav Sci.

[54] Hayes SC (2021). Acceptance and Commitment Therapy exceeds 600 randomized controlled trials. Association for Contextual Behavioral Science.

[55] Lloyd J, Bond FW, Flaxman PE (2013). The value of psychological flexibility: examining psychological mechanisms underpinning a cognitive behavioural therapy intervention for burnout. Work Stress.

[56] Vilardaga R, Luoma JB, Hayes SC, Pistorello J, Levin ME, Hildebrandt MJ, Kohlenberg B, Roget NA, Bond F (2011). Burnout among the addiction counseling workforce: the differential roles of mindfulness and values-based processes and work-site factors. J Subst Abuse Treat.

[57] Veage S, Ciarrochi J, Deane FP, Andresen R, Oades LG, Crowe TP (2014). Value congruence, importance and success and in the workplace: links with well-being and burnout amongst mental health practitioners. J Context Behav Sci.

[58] Puolakanaho A, Tolvanen A, Kinnunen SM, Lappalainen R (2020). A psychological flexibility -based intervention for Burnout: a randomized controlled trial. J Context Behav Sci.

[59] Gloster AT, Meyer AH, Lieb R (2017). Psychological flexibility as a malleable public health target: evidence from a representative sample. J Context Behav Sci.

[60] Ruiz FJ, Odriozola-González P (2017). The predictive and moderating role of psychological flexibility in the development of job burnout. Univ Psychol.

[61] Bond FW, Bunce D (2003). The role of acceptance and job control in mental health, job satisfaction, and work performance. J Appl Psychol.

[62] Arslan G, Allen K (2021). Exploring the association between coronavirus stress, meaning in life, psychological flexibility, and subjective well-being. Psychol Health Med.

[63] Bond FW, Bunce D (2000). Mediators of change in emotion-focused and problem-focused worksite stress management interventions. J Occupat Health Psychol.

[64] Bond FW, Hayes SC, Barnes-Holmes D (2006). Psychological flexibility, ACT, and organizational behavior. J Organ Behav Manag.

[65] Brinkborg H, Michanek J, Hesser H, Berglund G (2011). Acceptance and Commitment Therapy for the treatment of stress among social workers: a randomized controlled trial. Behav Res Ther.

[66] Flaxman PE, Bond FW (2010). A randomised worksite comparison of Acceptance and Commitment Therapy and stress inoculation training. Behav Res Ther.

[67] Frögéli E, Djordjevic A, Rudman A, Livheim F, Gustavsson P (2016). A randomized controlled pilot trial of acceptance and commitment training (ACT) for preventing stress-related ill health among future nurses. Anxiety Stress Coping.

[68] Ost LG (2014). The efficacy of Acceptance and Commitment Therapy: an updated systematic review and meta-analysis. Behav Res Ther.

[69] Kinnunen SM, Puolakanaho A, Tolvanen A, Mäkikangas A, Lappalainen R (2019). Does mindfulness-, acceptance-, and value-based intervention alleviate burnout?—A person-centered approach. Int J Stress Manag.

[70] Kinnunen SM, Puolakanaho A, Mäkikangas A, Tolvanen A, Lappalainen R (2020). Does a mindfulness-, acceptance-, and value-based intervention for burnout have long-term effects on different levels of subjective well-being?. Int J Stress Manag.

[71] Solms L, van Vianen AE, Theeboom T, Koen J, de Pagter AP, de Hoog M (2019). Keep the fire burning: a survey study on the role of personal resources for work engagement and burnout in medical residents and specialists in the Netherlands. BMJ Open.

[72] Regehr C, Glancy D, Pitts A, LeBlanc VR (2014). Interventions to reduce the consequences of stress in physicians: a review and meta-analysis. J Nerv Ment Dis.

[73] Berjot S, Altintas E, Grebot E, Lesage F (2017). Burnout risk profiles among French psychologists. Burnout Res.

[74] Villatte JL, Vilardaga R, Villatte M, Vilardaga JC, Atkins DC, Hayes SC (2016). Acceptance and Commitment Therapy modules: differential impact on treatment processes and outcomes. Behav Res Ther.

[75] Levin ME, Haeger J, Cruz RA (2018). Tailoring Acceptance and Commitment Therapy skill coaching in the moment through smartphones: results from a randomized controlled trial. Mindfulness.

[76] Obregon M, Luo J, Shelton J, Blevins T, MacDowell M (2020). Assessment of burnout in medical students using the Maslach Burnout Inventory-Student Survey: a cross-sectional data analysis. BMC Med Educ.

[77] Schaufeli WB, Martínez IM, Pinto AM, Salanova M, Bakker AB (2016). Burnout and engagement in university students. J Cross Cult Psychol.

[78] Keyes CL (2005). Mental illness and/or mental health? Investigating axioms of the complete state model of health. J Consult Clin Psychol.

[79] Keyes CL (2009). Brief description of the mental health continuum short form (MHC-SF). HSPH Harvard Education.

[80] Levin ME, Haeger JA, Pierce BG, Twohig MP (2017). Web-based Acceptance and Commitment Therapy for mental health problems in college students: a randomized controlled trial. Behav Modif.

[81] Seidler D, Stone B, Clark BE, Koran J, Drake CE (2020). Evaluating the factor structure of the Multidimensional Psychological Flexibility Inventory: an independent replication and extension. J Context Behav Sci.

[82] Perski O, Blandford A, West R, Michie S (2016). Conceptualising engagement with digital behaviour change interventions: a systematic review using principles from critical interpretive synthesis. Transl Behav Med.

[83] Lovibond SH, Lovibond PF, Psychology Foundation of Australia (1995). Manual for the depression anxiety stress scales. Psychology Foundation Monograph.

[84] Henry JD, Crawford JR (2005). The short-form version of the Depression Anxiety Stress Scales (DASS-21): construct validity and normative data in a large non-clinical sample. Br J Clin Psychol.

[85] Reeve A, Tickle A, Moghaddam N (2018). Are Acceptance and Commitment Therapy-based interventions effective for reducing burnout in direct-care staff? A systematic review and meta-analysis. Ment Health Rev J.

[86] Larmar S, Wiatrowski S, Lewis-Driver S (2014). Acceptance and Commitment Therapy: an overview of techniques and applications. J Serv Sci Manag.

[87] Stockton D, Kellett S, Berrios R, Sirois F, Wilkinson N, Miles G (2019). Identifying the underlying mechanisms of change during Acceptance and Commitment Therapy (ACT): a systematic review of contemporary mediation studies. Behav Cogn Psychother.

[88] Levin ME, Haeger J, Pierce B, Cruz RA (2017). Evaluating an adjunctive mobile app to enhance psychological flexibility in Acceptance and Commitment Therapy. Behav Modif.

[89] Baumel A, Muench F, Edan S, Kane JM (2019). Objective user engagement with mental health apps: systematic search and panel-based usage analysis. J Med Internet Res.

[90] Kim E, Mallett R, Hrabok M, Yang YA, Moreau C, Nwachukwu I, Kravtsenyuk M, Abba-Aji A, Li D, Agyapong VI (2020). Reducing burnout and promoting health and wellness among medical students, residents, and physicians in alberta: protocol for a cross-sectional questionnaire study. JMIR Res Protoc.

