# Correction: Assessing the Efficacy of an Individualized Psychological Flexibility Skills Training Intervention App for Medical Student Burnout and Well-being: Protocol for a Randomized Controlled Trial

**DOI:** 10.2196/37798

**Published:** 2022-03-16

**Authors:** Elizabeth Ditton, Brendon Knott, Nicolette Hodyl, Graeme Horton, Frederick Rohan Walker, Michael Nilsson

**Affiliations:** 1 Centre for Rehab Innovations University of Newcastle Callaghan Australia; 2 Hunter Medical Research Institute New Lambton Heights Australia; 3 College of Health, Medicine and Wellbeing University of Newcastle Callaghan Australia; 4 Contextual Interventions Newcastle Australia; 5 Lee Kong Chian School of Medicine Nanyang Technological University Singapore Singapore

In “Assessing the Efficacy of an Individualized Psychological Flexibility Skills Training Intervention App for Medical Student Burnout and Well-being: Protocol for a Randomized Controlled Trial” (JMIR Res Protoc 2022;11(2):e32992) the authors noted two errors.

First, in the originally published article a footnote appeared below Table 1 as follows:

^a^N/A: not applicable.

This was deleted in the correction as it did not correspond to any values in Table 1.

Second, in the originally published article, the duration of the trial intervention was reported as 5 weeks. The actual duration of the intervention was 8 weeks.

The value appeared incorrectly in the following 7 instances:

1. Abstract; Methods

Participants in the individualized and nonindividualized intervention arms will have 5 weeks to access the app, which includes a PF concepts training session (stage 1) and access to short PF skill activities on demand (stage 2).

This has been corrected as follows:

Participants in the individualized and nonindividualized intervention arms will have 8 weeks to access the app, which includes a PF concepts training session (stage 1) and access to short PF skill activities on demand (stage 2).

2. Methods; Data Collection Tools and Procedures

Data will be collected at two time points: T1 (baseline) and T2 following the completion of the app-based intervention, commencing 5 weeks after baseline

This has been corrected as follows:

Data will be collected at two time points: T1 (baseline) and T2 (following the completion of the app-based intervention, commencing 8 weeks after baseline).

3. Methods; Intervention Stages

Participants who are allocated to the individualized and nonindividualized groups will have access to the 2-stage app for 5 weeks.

This has been corrected as follows:

Participants who are allocated to the individualized and nonindividualized groups will have access to the 2-stage app for 8 weeks.

4. Methods section; Intervention Stages

Participants may complete as many activities as they choose, but will be asked to complete at least four stage 2 skill activities during their 5-week period of access to the app.

This has been corrected as follows:

Participants may complete as many activities as they choose, but will be asked to complete at least four stage 2 skill activities during their 8-week period of access to the app.

5. Methods; Study Design; Figure 1

Access stage 2: individualized program (5-week access period)

This has been corrected as follows:

Access stage 2: individualized program (8-week access period)

6. Methods; Study Design; Figure 1

Access stage 2: nonindividualized program (5-week access period)

This has been corrected as follows:

Access stage 2: nonindividualized program (8-week access period)

7. Methods; Study Design; Figure 1

Complete t_2_ assessments (t=5 weeks)

This has been corrected as follows:

Complete t_2_ assessments (t=8 weeks)

The corrected version of Figure 1 is included below. The originally published version of Figure 1 is attached as Multimedia Appendix 1.

The correction will appear in the online version of the paper on the JMIR Publications website on March 16, 2022, together with the publication of this correction notice. Because this was made after submission to PubMed, PubMed Central, and other full-text repositories, the corrected article has also been resubmitted to those repositories.

**Figure 1:**
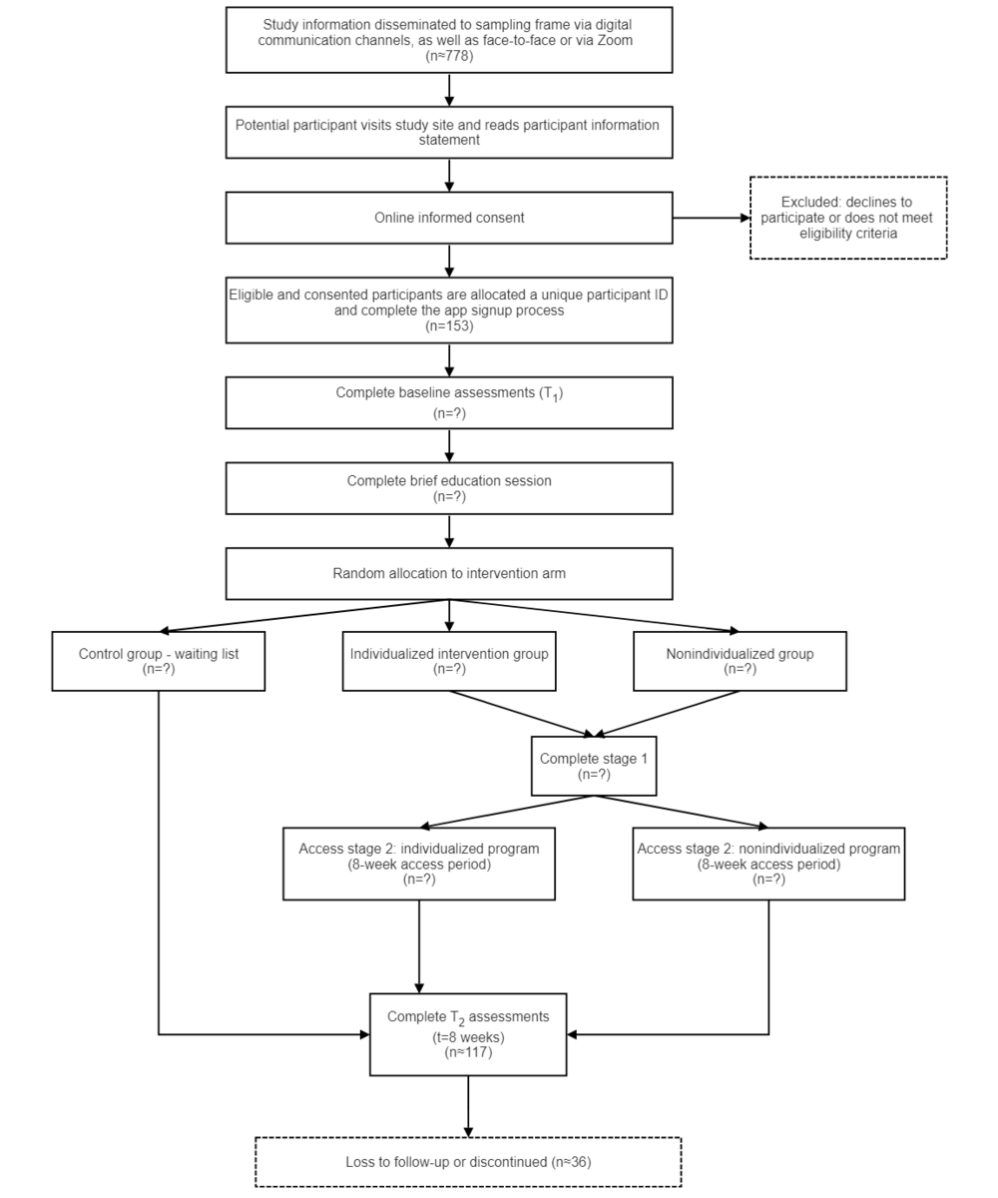
Participant timeline.

## Appendix

Multimedia Appendix 1 Original Figure 1.

