# Correction: Assessing the Efficacy of an Individualized Psychological Flexibility Skills Training Intervention App for Medical Student Burnout and Well-being: Protocol for a Randomized Controlled Trial

**DOI:** 10.2196/40684

**Published:** 2022-07-11

**Authors:** Elizabeth Ditton, Brendon Knott, Nicolette Hodyl, Graeme Horton, Frederick Rohan Walker, Michael Nilsson

**Affiliations:** 1 Centre for Rehab Innovations University of Newcastle Callaghan Australia; 2 Hunter Medical Research Institute New Lambton Heights Australia; 3 College of Health, Medicine and Wellbeing University of Newcastle Callaghan Australia; 4 Contextual Interventions Newcastle Australia; 5 Lee Kong Chian School of Medicine Nanyang Technological University Singapore Singapore

In “Assessing the Efficacy of an Individualized Psychological Flexibility Skills Training Intervention App for Medical Student Burnout and Well-being: Protocol for a Randomized Controlled Trial” (JMIR Res Protoc 2022;11(2):e32992), the authors made the following update.

On March 17, 2022, the authors had published a corrigendum [1] to change the reported intervention duration from 5 weeks to 8 weeks. However, the intervention duration reported in the originally published article was correct. The current corrigendum restores the reported intervention duration to 5 weeks with the following changes:

1. In the *Methods* section of the *Abstract*, a statement appeared as follows:

Participants in the individualized and nonindividualized intervention arms will have 8 weeks to access the app, which includes a PF concepts training session (stage 1) and access to short PF skill activities on demand (stage 2).

This has been corrected as follows:

Participants in the individualized and nonindividualized intervention arms will have 5 weeks to access the app, which includes a PF concepts training session (stage 1) and access to short PF skill activities on demand (stage 2).

2. In the *Data Collection Tools and Procedures* section of *Methods*, a statement appeared as follows:

Data will be collected at two time points: T1 (baseline) and T2 (following the completion of the app-based intervention, commencing 8 weeks after baseline).

This has been corrected as follows:

Data will be collected at two time points: T1 (baseline) and T2 (following the completion of the app-based intervention, commencing 5 weeks after baseline).

3. In the *Intervention Stages* section of *Methods*, a statement appeared as follows:

Participants who are allocated to the individualized and nonindividualized groups will have access to the 2-stage app for 8 weeks.

This has been corrected as follows:

Participants who are allocated to the individualized and nonindividualized groups will have access to the 2-stage app for 5 weeks.

4. In the *Intervention Stages* section of *Methods*, a statement appeared as follows:

Participants may complete as many activities as they choose, but will be asked to complete at least four stage 2 skill activities during their 8-week period of access to the app.

This has been corrected as follows:

Participants may complete as many activities as they choose, but will be asked to complete at least four stage 2 skill activities during their 5-week period of access to the app.

5. Following the previous corrigendum [1], Figure 1 was altered to reflect the intervention duration of 8 weeks. The present corrigendum updated Figure 1 as follows:

**Figure 1:**
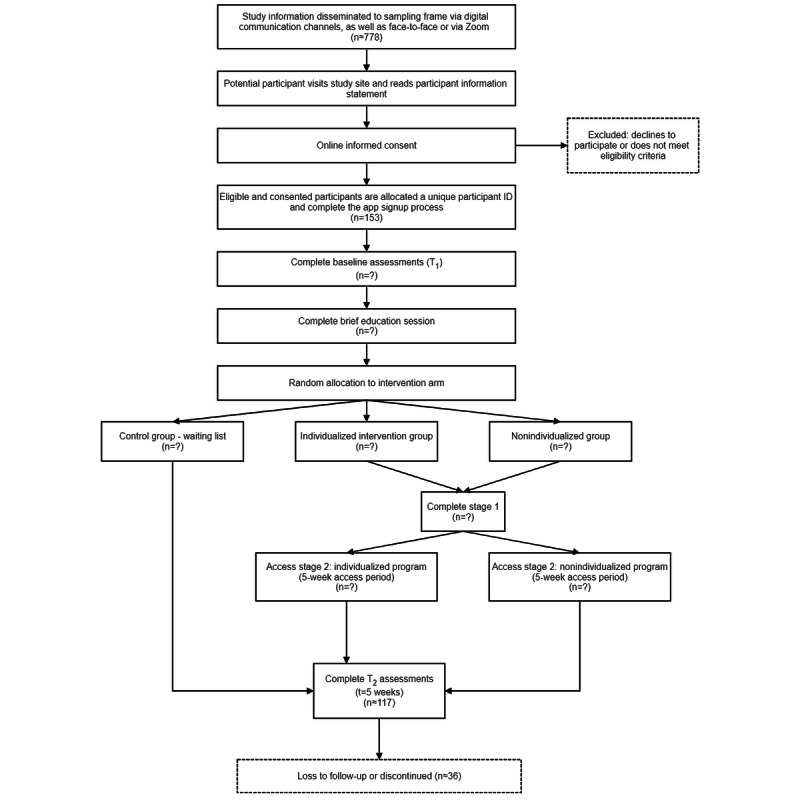
Participant timeline.

The correction will appear in the online version of the paper on the JMIR Publications website on July 11, 2022, together with the publication of this correction notice. Because this was made after submission to PubMed, PubMed Central, and other full-text repositories, the corrected article has also been resubmitted to those repositories.
