# Supplementary material for: Assessing the Efficacy of an Individualized Psychological Flexibility Skills Training Intervention App for Medical Student Burnout and Well-being: Protocol for a Randomized Controlled Trial
Source: JMIR Res Protoc. 2022 Feb 4;11(2):e32992. doi: 10.2196/32992 (PMC8857701; doi:10.2196/32992)
Supplement: Multimedia Appendix 1 [file resprot_v11i2e32992_app1.docx]

**Appendix 1.** Aims and example activities for each psychological flexibility skill set

| Psychological flexibility process | Aims | Example activity |
| --- | --- | --- |
| Present-moment awareness (mindfulness) | Train participants to engage in purposeful awareness of their present moment experiences (internal and external). Enhance participants’ awareness of how their direct experiences influence their choices and behaviors. | Participants are presented with a brief mindfulness activity in which they are guided to bring their attention to specific aspects of their present-moment experience (eg, sounds, physical sensations, and breathing). |
| Defusion | Facilitate participants’ awareness of the difference between their direct experiences and their *thoughts* about their direct experiences. Help participants recognize the impact of responding to their thoughts as though they are the same as their experiences (“fusion”), and the consequences of fusion with unhelpful thought content. Facilitate the identification of fusion as it occurs. Train participants to respond flexibly to unhelpful thoughts, through changing the contexts in which they experience their thoughts. | Participants are asked to identify a thought they are currently having difficulty with. They are guided through a brief exercise that helps them “step back” (or “defuse”) from that thought by experiencing the same thought in a different context. For example, visualizing the words on a movie screen off at a far distance; singing the thought to a familiar tune; or the “leaves on a stream” activity. |
| Self-as-context (perspective-taking) | Train participants to recognize that their thoughts and emotions do not define their identity but are aspects of their experience. Help participants adopt a perspective that allows them to take a step back from their internal experiences to expand their behavioral options and facilitate more effective and flexible actions. | Participants are asked to bring their attention to their thoughts. They are prompted to notice the nature of their thoughts (eg, visual, auditory, and words) and to observe where these thoughts are located (eg, in their head, above them, or in their body). Participants are then directed to notice that there is a part of themselves experiencing the thoughts and a part of themselves observing that experience. (ie, “You are not your thoughts, your thoughts are transient experiences”). |
| Acceptance | Help participants identify whether they engage in unhelpful avoidance. Facilitate more flexible and adaptive ways of responding to unavoidable internal discomfort. Help participants identify what they can control and what they cannot, and take effective action in both contexts. | Participants are trained to observe and experience an uncomfortable sensation (eg, an itch) with openness, curiosity, and willingness. They are prompted to focus on the components comprising the sensation (eg, size, movement, shape, and temperature) and to notice the transience of these internal experiences. |
| Values | Help participants identify their own personal sources of meaning, purpose, and vitality. Facilitate the daily connection with an individual’s values through action and awareness or reflection. Facilitate a conscious connection between a participant’s chosen behaviors and their values. Help participants understand how values can be used as a higher-order motivating influence over their behavior, particularly in situations in which they may have limited control. | Participants are presented with the list of the core values they identified during stage 1 of the app. They are encouraged to choose one of the values and briefly reflect on how they have acted in alignment with that value during the day. |
| Committed action | Help participants use skills and resources developed during the intervention, to take action toward what is important in their lives. Focus on establishing willingness to pursue what is personally important, even when there are internal and external challenges (ie, to help people do what matters even when discomfort is present). Train participants to leverage their awareness to make more effective and consciously-driven choices regarding their direct experiences and personal values. | Participants are presented with the list of the core values they identified during stage 1 of the app. They are encouraged to choose one value that feels most important to them in that moment and to take one small and achievable action toward that value during the day. |
