# Supplementary figures and images for: Correction: Assessing the Efficacy of an Individualized Psychological Flexibility Skills Training Intervention App for Medical Student Burnout and Well-being: Protocol for a Randomized Controlled Trial
Source: JMIR Res Protoc. 2022 Mar 16;11(3):e37798. doi: 10.2196/37798 (PMC8968621; doi:10.2196/37798)

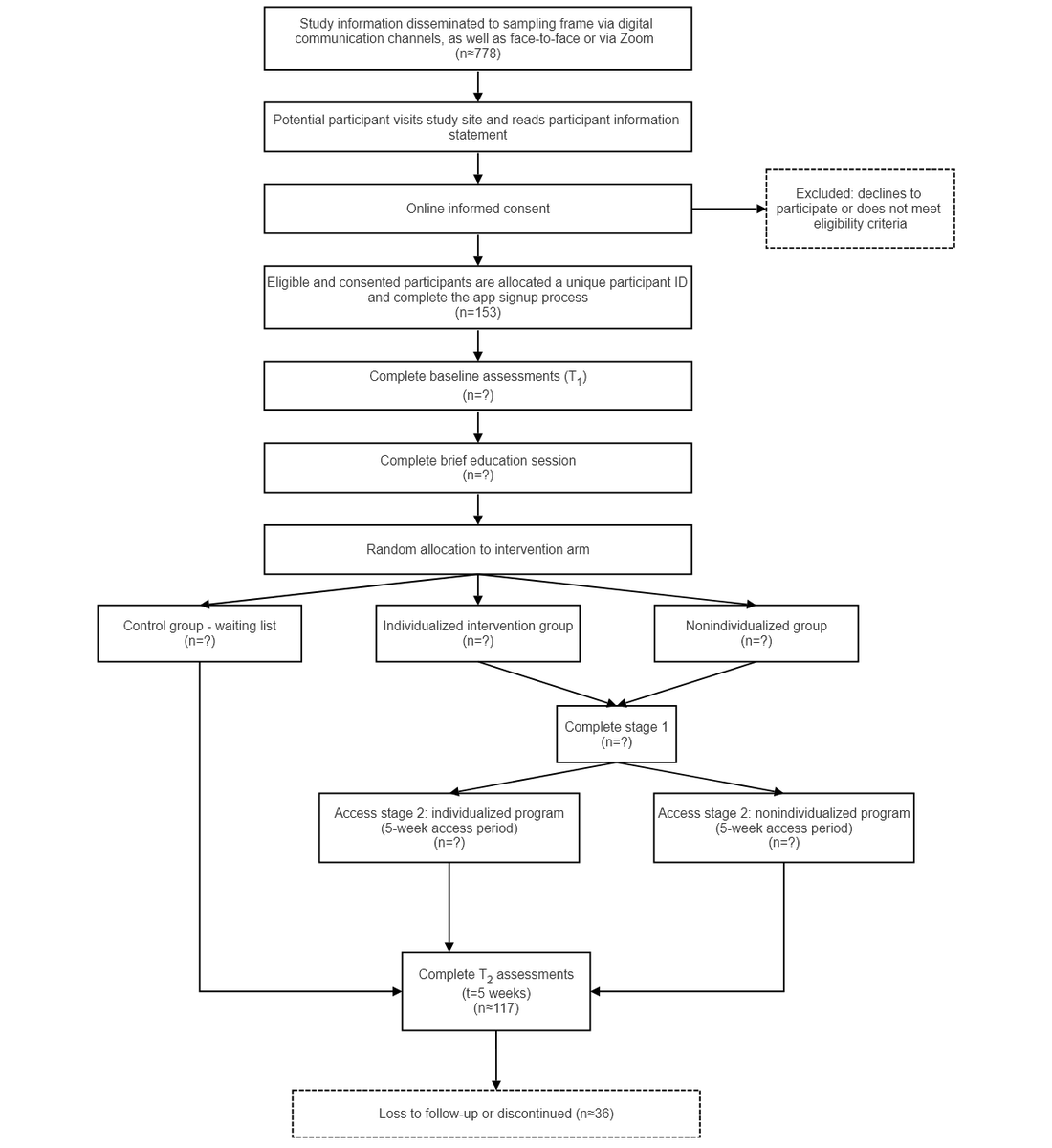

Supplement: Multimedia Appendix 1 [file resprot_v11i3e37798_app1.png]
